# MiR-302b Suppresses Osteosarcoma Cell Migration and Invasion by Targeting Runx2

**DOI:** 10.1038/s41598-017-13353-9

**Published:** 2017-10-17

**Authors:** Yuanlong Xie, Wenchao Sun, Zhouming Deng, Xiaobin Zhu, Chao Hu, Lin Cai

**Affiliations:** Department of Orthopedics, Zhongnan Hospital of Wuhan University, Wuhan, People’s Republic of China

## Abstract

Osteosarcoma patients with lung metastasis and local invasion remain challenging to treat despite the significant contribution of the combination of surgery and neo-adjuvant chemotherapy. Our previous microarray study demonstrated that miR-302b had significantly lower expression in osteosarcoma cell lines than in osteoblast cell lines. In the present study, we further elucidated the role of miR-302b in regulating the migration and invasiveness of osteosarcoma. MiR-302b expression was markedly down-regulated in osteosarcoma cell lines and clinical tumour tissues. Lower levels of miR-302b expression were significantly associated with metastasis and high pathological grades. A functional study demonstrated that over-expression of miR-302b suppressed tumour cell proliferation, invasion and migration *in vitro* and *in vivo*. Runx2 was identified as a direct target gene for miR-302b by bioinformatics analysis and dual-luciferase reporter gene assay. Moreover, over-expression of miR-302b induced down-regulation of Runx2, OPN, MMP-2, MMP-9, MMP-12, MMP-14, and VEGF in 143B cells. Exogenous expression of Runx2 partially rescued the inhibitory effect of miR-302b on the invasion and migration activity of 143B osteosarcoma cells. Taken together, our results indicate that miR-302b functions as a tumour repressor in the invasion and migration of osteosarcoma by directly downregulating Runx2 expression and may be a potential therapeutic target for osteosarcoma.

## Introduction

Osteosarcoma arising from bone is the most common primary malignant tumour in children, adolescents, and young adults^1^. Despite the significant contribution of the combination of surgery and neo-adjuvant chemotherapy, the clinical outcomes and prognosis of patients suffering from osteosarcoma have made little progress in the past ten years^2^. Metastasis is one of the most intricate aspects of osteosarcoma. Osteosarcoma patients with lung metastasis mostly became unable to undergo surgery, leading to a 5-year survival rate of under 30%^3^. In contrast, the 5-year survival rate of patients without distant metastasis is over 60%^4^. The underlying molecular mechanisms of carcinogenesis and metastatic development remain unclarified. Accumulating evidence has shown that short non-coding RNA known as microRNAs (miRNAs) are involved in the progression and metastasis of osteosarcoma by regulating target mRNAs via binding to their 3′-untranslated regions (UTRs) in a sequence-specific pattern^5,6^. MiRNAs dysfunction play significant roles in several biological processes, including cell proliferation, differentiation, apoptosis, cell cycle, migration and invasion^7^. For example, reduction of miR-143 increases osteosarcoma cell invasion by targeting MMP-13^8^. In addition, miR-20a promotes the metastatic potential of osteosarcoma cells by regulating the Fas/FasL system^9^.

Our previous study demonstrated by miRNA microarrays and bioinformatic analysis that several miRNAs are differentially expressed between osteosarcoma and osteoblast cell lines^10^. MiR-302b, one of the 268 dysregulated miRNAs, is significantly under-expressed in osteosarcoma cell lines compared with osteoblast cell lines^10^. Furthermore, miR-302b can restrain the proliferation of osteosarcoma cells; promote cell apoptosis by regulating Akt/pAkt, Bcl-2, and Bim; and promote cell cycle arrest by attenuating the levels of cyclin D1 and CDKs^11^. In addition, evidence shows that miR-302b suppresses cell invasion and metastasis by directly targeting AKT2 in human hepatocellular carcinoma cells^12^. However, the potential function of miR-302b in osteosarcoma metastasis remains obscure.

In the current study, we explored the potential function of miR-302b in osteosarcoma cell invasion and migration. First, we examined the expression of miR-302b in osteosarcoma tissue and the relationship between miR-302b and clinical characteristics of osteosarcoma patients. Moreover, we investigated the potential role of miR-302b in the cell proliferation, invasion, and migration of osteosarcoma cell lines. Next, we explored the underlying molecular mechanism of the function of miR-302b in osteosarcoma by bioinformatics analysis and rescue experiments. Finally, the potential role of miR-302b in osteosarcoma was further demonstrated in a nude mouse model. The present study provided a deeper understanding of miR-302b in the development and progression of osteosarcoma.

## Results

### The relationship between miR-302b and clinical characteristics of osteosarcoma patients

**Figure 1:**
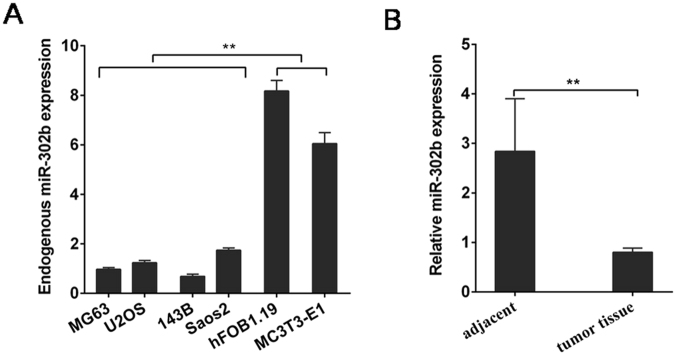
Dysregulated miR-302b in osteosarcoma tissues and cells. (**A**) qRT- PCR was used to analyse miR-302b expression in osteosarcoma cells and osteoblastic cells. (**B**) qRT-PCR was performed to examine miR-302b expression in 31 pairs of tissue samples consisting of human osteosarcoma and adjacent normal bone tissues. (*P < 0.05, **P < 0.01).

Initially, quantitative real-time PCR (qRT-PCR) was used to detect the miR-302b expression levels of several osteosarcoma cell lines (MG-63,U2OS,143B,Saos2) and two osteoblastic cell lines (hFOB1.19, MC3T3-E1). The results showed that miR-302b expression levels in the MG-63,U2OS,143B,and Saos2 cell lines were significantly lower than those in the two osteoblastic cell lines (hFOB1.19, MC3T3-E1) (Fig. 1A).Then, detection of miR-302b expression was performed using qRT-PCR in 31 pairs of human primary osteosarcoma tumours and adjacent normal bone tissues. The results showed that the mean level of miR-302b was lower in osteosarcoma tissue than that in the adjacent normal bone tissues (Fig. 1B). To explore the clinicopathologic significance of miR-302b variation, we quantified the levels of miR-302b in 31 pairs of osteosarcoma tumours using qRT-PCR. A low-expression (≤median) group and a high-expression (>median) group were defined using the median value (0.81) of miR-302b expression as a cut-off point. As shown in Table 1, low expression of miR-302b was significantly correlated with metastasis and high pathological grades (P < 0.05), whereas no significant correlation was observed for other parameters. These results showed that downregulation of miR-302b contributed to OS pathogenesis.

**Table 1:** Correlation between the expression of miR-302b and clinicopathological data.

Clinical feature	miR-302b expression			
	high (å > 0.81)	low (≤0.81)	*t/X* ^2^	*P*
Age (years)	18.80 ± 0.83	16.94 ± 0.99	1.437	0.162
Gender				
Male	10	8	0.267	0.605
Female	6	7		
Primary site				
Femur	6	5	0.535	0.911
Tibia	5	5		
Humerus	2	1		
Other	3	4		
Metastasis				
Yes	9	13	4.454	0.035
No	7	2		
TNM grade				
high grade	8	14	5.444	0.020
low grade	8	1		

### Overexpression of miR-302b suppresses cell proliferation of 143B and MG-63 cells

**Figure 2:**
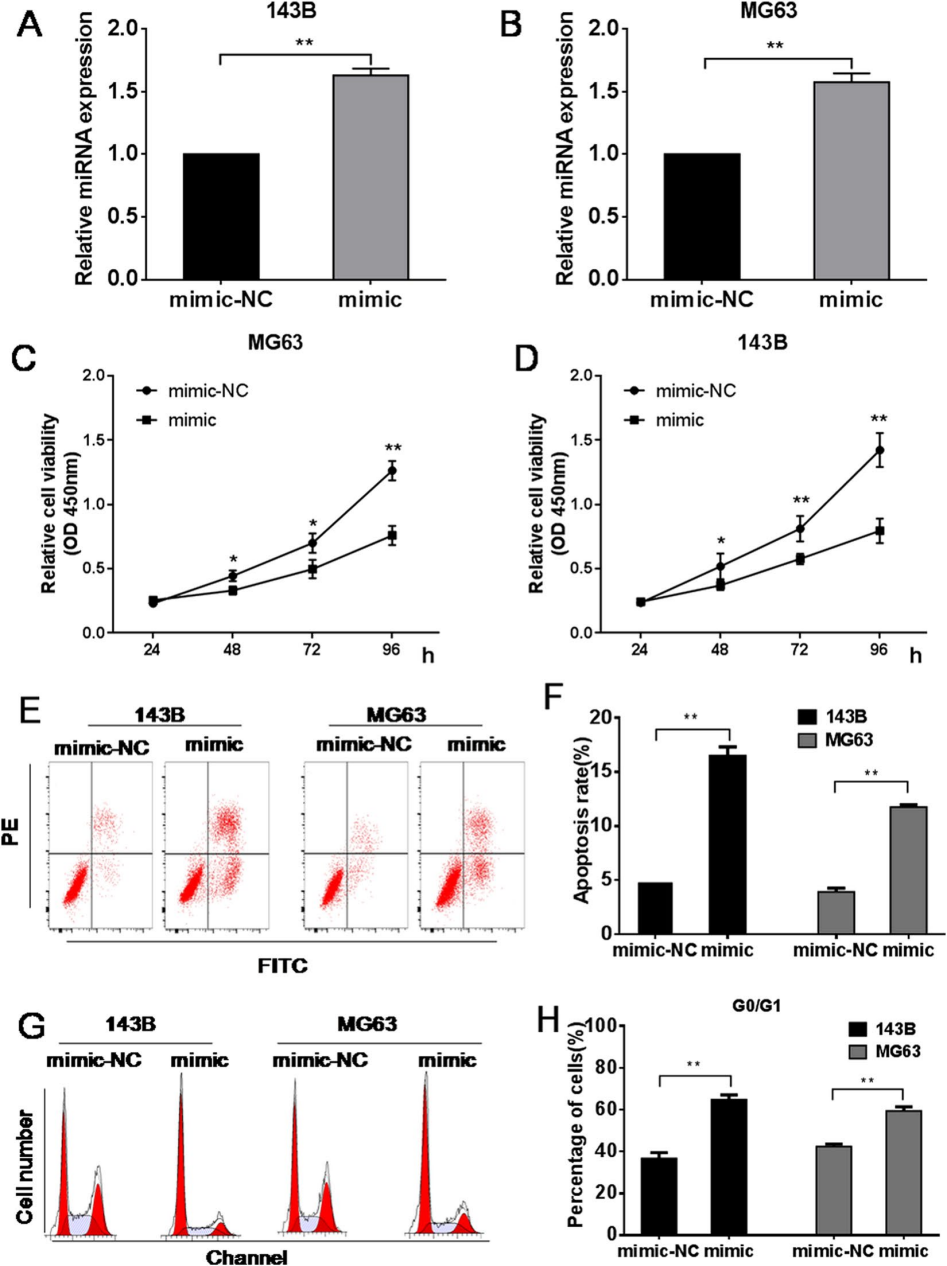
miR-302b suppresses the proliferation and induces cell apoptosis and cycle arrest of 143B/MG-63 cells. (**A** and **B**) qRT-PCR analysis of miR-302b in 143B, MG63 cells transfected with miR-302b mimic (mimic), or miR-302b negative control (mimic-NC) as a control. (**C**) The effect of miR-302b mimics on 143B cell proliferation was detected with CCK-8 assays at 24, 48, 72, and 96 hours. (**D**) The effect of miR-302b mimics on MG-63 cell proliferation was detected with CCK-8 assays at 24, 48, 72, and 96 hours. (**E**,**F**) 143B/MG63 cells were transfected with miR-302b mimic, or mimic-NC as control. The cell apoptosis profiles were detected using flow cytometry. (**E**) A representative image of FACS plot. The FACS plot shows the cells in early (bottom right quadrant) and late apoptotic states (upper right quadrant). Viable cells are double negative (bottom left quadrant). (**F**) The results of a quantitative analysis. (**G** and **H**) 143B/MG63 cells were transfected with miR-302b mimic, or mimic-NC as control. The cell cycle profiles were detected using flow cytometry. (**G**) A representative image. (**H**) The results of a quantitative analysis. The results are expressed as the means ± SD of three independent experiments (NC: negative control, *P < 0.05, **P < 0.01).

To explore the potential role of miR-302b in osteosarcoma, we transiently transfected MG-63 and 143B osteosarcoma cells with miR-302b mimics and verified the miR-302b expression by qRT-PCR (Fig. 2A,B). Data from a CCK-8 assay showed that over-expression of miR-302b significantly suppressed the proliferation of 143B and MG-63 cells (Fig. 2C,D). Apoptosis and cell cycle distribution were examined to determine whether they accompanied the observed inhibition of cell growth. Over-expression of miR-302b increased cell apoptosis both in 143B cells and in MG-63 cells (Fig. 2E,F). As shown in Fig. 2G,H, the percentages of 143B and MG-63 osteosarcoma cells in G0/G1 significantly increased after the miR-302b transfection (P < 0.05). Our results suggested that miR-302b could induce cell apoptosis and cell cycle arrest, which might contribute to the inhibition of cell proliferation.

### Overexpression of miR-302b suppresses migration and invasion of 143B and MG-63 cells

**Figure 3:**
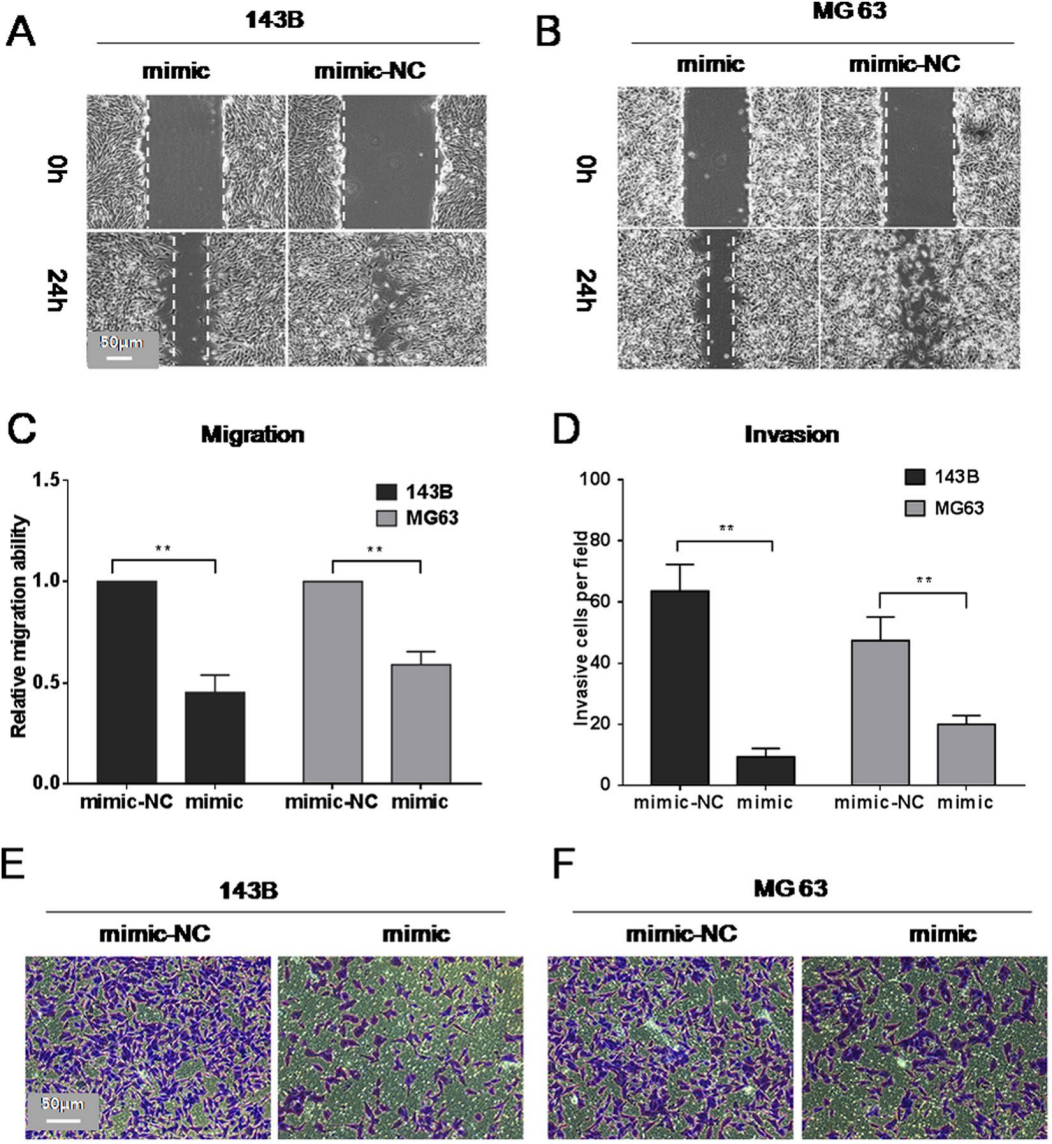
miR-302b suppresses the migration and invasion activity of 143B/MG-63 cells. (**A** and **B**) The effects of miR-302b mimics on the invasion ability of 143B and MG-63 cells were determined by a wound healing assay. (**C**) Relative migration ability was measured by the equation described in Materials and Methods. (**D**) Number of invasive cells was counted under a microscope in each group. (**E** and **F**) Representative results of a transwell assay detecting the effect of miR-302b mimics on 143B and MG-63 cells. (NC: negative control, *P < 0.05, **P < 0.01).

Further investigation was conducted on whether miR-302b inhibits the migration and invasion capabilities of osteosarcoma. The data from the wound healing assay showed that over-expression of miR-302b significantly suppressed the migration of 143B and MG-63 cells (Fig. 3A,B). The distance of cell migration was calculated to quantitatively determine the migration ability of the cells (Fig. 3C). Consistently, over-expression of miR-302b suppressed cell invasion of 143B and MG-63, which was determined using transwell assays (Fig. 3D,E,F).

### miR-302b targets the Runx2 gene in 143B cells

**Figure 4:**
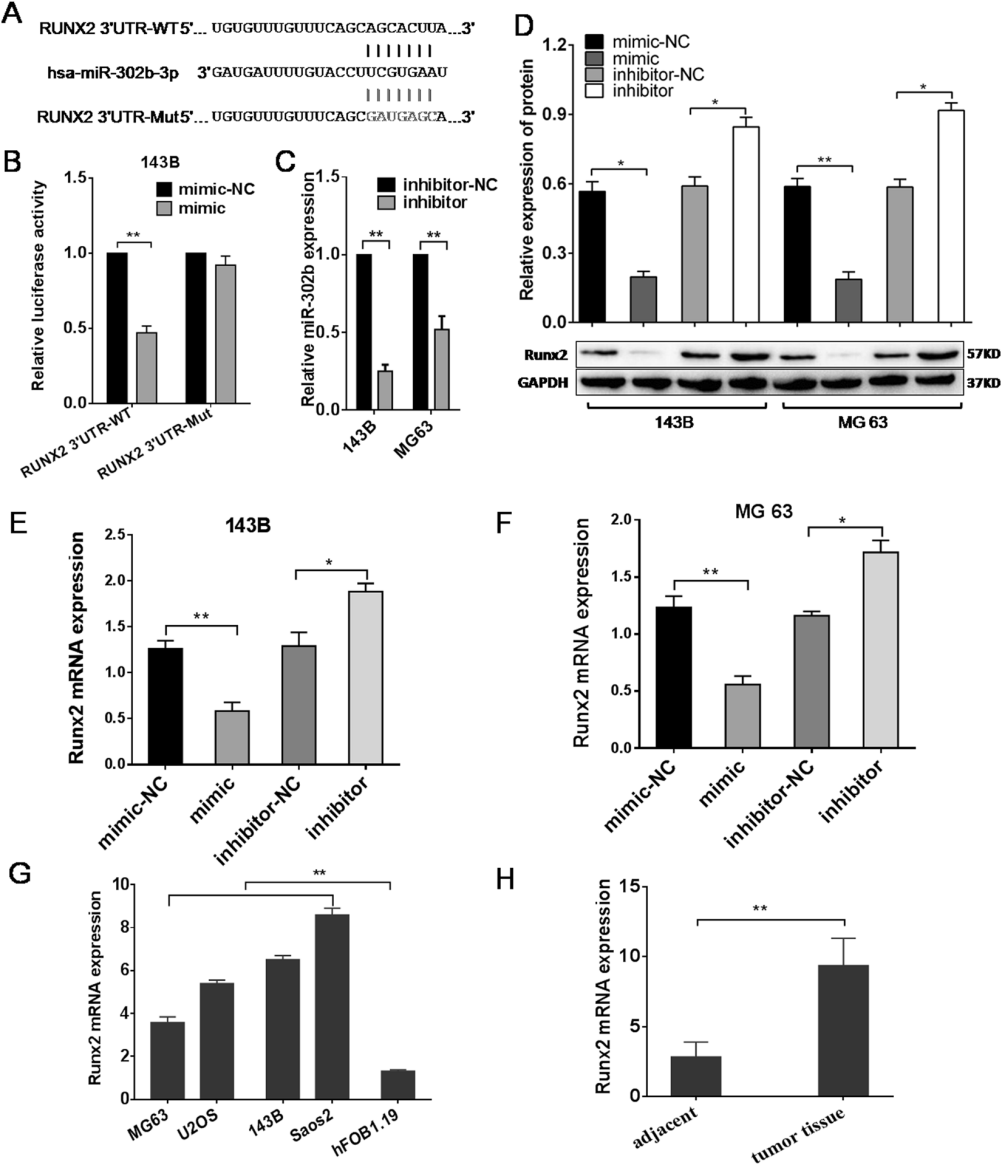
miR-302b targets the Runx2 gene. (**A**) Binding sites of miR-302b in the 3′-UTR region of Runx2. A mutant miR-302b binding site was generated in the complementary site for the seed region of miR-302b. (**B**) pmirGLO-Runx2-3′-UTR-wt or pmirGLO-Runx2-3′-UTR-mut was co-transfected with miR-302b or miR-ctrl in 143B cells, and the relative luciferase activity was measured. (**C**) qRT-PCR analysis of miR-302b in 143B, MG63 cells transfected with miR-302b inhibitor, or inhibitor-NC as a control. (**D**) Detection of Runx2 proteins in 143B and MG-63 cells after transfection with miR-302b mimics/inhibitor. (**E** and **F**) Detection of Runx2 mRNA in 143B and MG-63 cells after transfection with miR-302b mimics/inhibitor. (**G**) Detection of Runx2 mRNA in several osteosarcoma cells and osteoblast cell lines. (**H**) Detection of Runx2 mRNA in 31 pairs of osteosarcoma tissues and adjacent tissues. (NC: negative control, *P < 0.05, **P < 0.01).

Potential target genes were predicted by three online bioinformatics analysis software programs including miRWalk, miRbase and Target Scan. Runx2 containing a miR-302b binding site in the 3′-UTR was uniformly predicted by the software and Runx2 was selected for further experiments. A dual-luciferase reporter system containing a wild-type (AGCACUU) or mutant (GAUGAGC) 3′-UTR of Runx2 was used to verify whether miR-302b directly interacts with the 3′-UTR of Runx2 (Fig. 4A). 143B cells were co-transfected with miR-302b or miR-ctrl and with pmirGLO- Runx2-3′-UTR-wt or pmirGLO-Runx2-3′-UTR-mut. The data showed that the transfection with miR-302b significantly suppressed the luciferase activity of pmirGLO-Runx2-3′-UTR-wt but did not affect the luciferase activity of the pmirGLO reporter carrying the mutant Runx2-3′-UTR (Fig. 4B).MG-63 and 143B osteosarcoma cells were transiently transfected with miR-302b inhibitors and the down-regulation of miR-302b was verified by qRT-PCR (Fig. 4C). qRT-PCR and Western blotting were performed to detect the levels of mRNA and protein, respectively. Compared with the negative control group, the protein levels of Runx2 were significantly reduced or promoted in response to miR-302b mimics and miR-302b inhibitors, respectively, in 143B and MG63 cells (Fig. 4D). Additionally, the mRNA levels of Runx2 were significantly reduced or promoted respectively in response to miR-302b mimics and miR-302b inhibitors compared with the negative control group in 143B and MG63 cells (Fig. 4E,F). qRT-PCR was used exploring the expression levels of Runx2 in OS cells (MG-63,U2OS,143B,Saos2) relative to osteoblast cells (hFOB1.19) and 31 pairs OS tissue relative to adjacent tissue. The results showed that Runx2 mRNA expression levels in several osteosarcoma cell lines were significantly lower than those in the two osteoblastic cell lines (Fig. 4G). The mean expression level of Runx2 mRNA was lower in osteosarcoma tissue than in the adjacent normal bone tissues (Fig. 4H). These results indicated that the Runx2-3′-UTR carried the binding sites for miR-302b.

### MiR-302b and the target gene Runx2 share analogous molecular effects in 143B osteosarcoma cells

**Figure 5:**
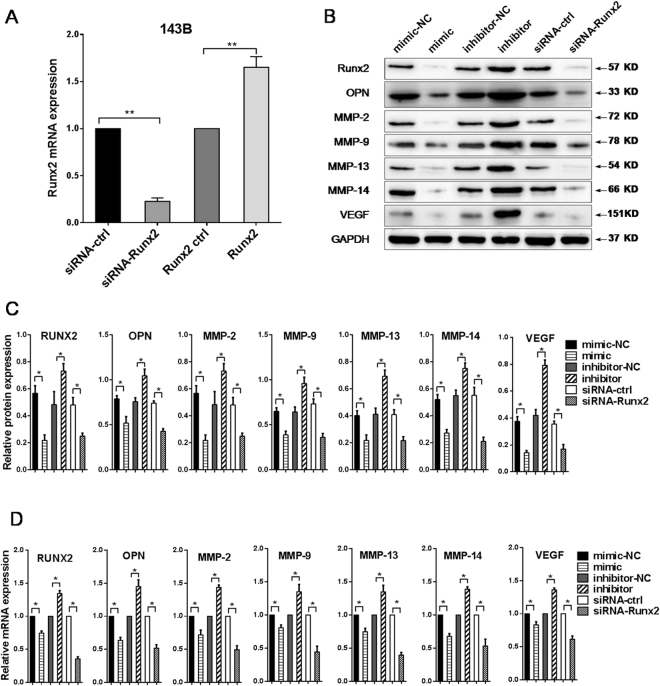
miR-302b and the target gene Runx2 share analogous molecular effects in 143B osteosarcoma cells. (**A**) qRT-PCR analysis of Runx2 in 143B cells transfected with siRNA-Runx2 or a Runx2 overexpression construct, with an siRNA control and an empty vector, respectively, as their controls. (**B**) Detection of Runx2, OPN, MMP-2, MMP-9, MMP-13, MMP-14 and VEGF proteins in 143B cells after transfection with miR-302b mimics/inhibitor or siRNA-Runx2. (**C**) Relative protein expression level in each group. (**D**) Detection of Runx2, OPN, MMP-2, MMP-9, MMP-13, MMP-14 and VEGF mRNA in 143B cells after transfection with miR-302b mimics/inhibitor or siRNA-Runx2. (*P < 0.05, **P < 0.01).

Runx2 expression was silenced by RNAi method to detect whether Runx2 was involved in the anti-tumour effect of miR-302b. First, the transfection rate of Runx2 over-expression vector, Runx2-siRNA were verified by qRT-PCR in 143B cells (Fig. 5A). As shown in Fig. 5B–D, miR-302b mimics suppressed the mRNA and protein levels of Runx2, OPN, MMP-2, MMP-9, MMP-13, MMP-14 and VEGF compared with the negative control group in 143B cells. In addition, an miR-302b inhibitor promoted the mRNA and protein levels of these gene products, which are the essential regulators of cell migration and invasion. Notably, Runx2 siRNA could inhibit the mRNA and protein level of Runx2, OPN, MMP-2, MMP-9, MMP-13, MMP-14 and VEGF, similar to the suppressive effect mediated by miR-302b mimics in 143B cells. These data suggested that miR-302b and the target gene Runx2 share analogous molecular effects in 143B osteosarcoma cells.

### Overexpression of Runx2 rescues the inhibitory effect of miR-302b on 143B cells

**Figure 6:**
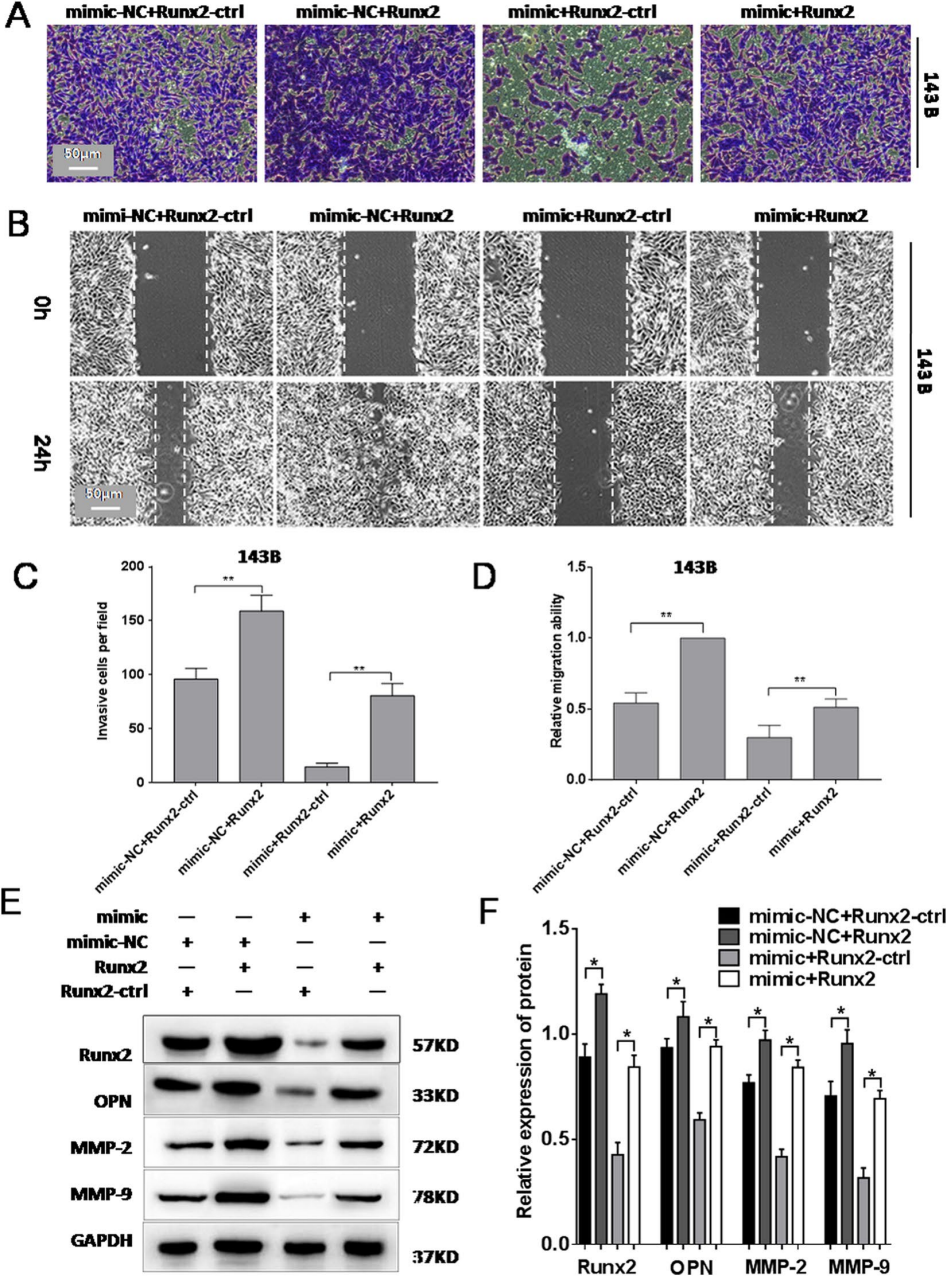
Overexpression of Runx2 rescued the inhibiting effect of miR-302b on 143B osteosarcoma cells. (**A**) miR-302b mimic (miR-302b) or miR-302b negative control (miR-ctrl) and Runx2 overexpression construct (Runx2) or an empty vector (Runx2-ctrl) were cotransfected into 143B/MG-63 cells, and the representative results of the transwell assay were shown. (**B**) Representative results of a wound healing assay in 143B cells. (**C**) number of invasive cells counted under a microscope in each group. (**D**) Relative migration ability of each group. (**E**) Detection of Runx2, OPN, MMP-2, and MMP-9 proteins in 143B after the transfection. (**F**) Relative protein expression level in each group. (*P < 0.05, **P < 0.01).

To further demonstrate the tumour suppressor function of miR-302b through targeting of Runx2, an miR-302b mimic (miR-302b) or miR-ctrl and a Runx2 over-expression vector (Runx2) or a negative control vector (Runx2-ctrl) were co-transfected into 143B cells. As shown in Fig. 6A–D, cell invasion and migration were up-regulated after the co-transfection of miR-302b and Runx2 compared with the co-transfection of miR-302b and Runx2-ctrl. In other words, over-expression of Runx2 counterbalanced the tumour suppressor effect of miR-302b mimics on cell invasion and migration in 143B cells. As shown in Fig. 6E,F, over-expression of Runx2 in 143B cells rescued the down-regulation of Runx2 protein expression level mediated by miR-302b mimics. Furthermore, the results showed that over-expression of Runx2 in 143B cells also rescued the miR-302b mimics mediated down-regulation of protein expression of OPN, MMP-2, and MMP-9. These results revealed that over-expression of Runx2 rescued the inhibitory effect of miR-302b on 143B osteosarcoma cells, which further demonstrated that miR-302b suppressed the 143B osteosarcoma cells by targeting Runx2.

### miR-302b inhibits orthotopic osteosarcoma tumour growth and lung metastasis in nude mice

**Figure 7:**
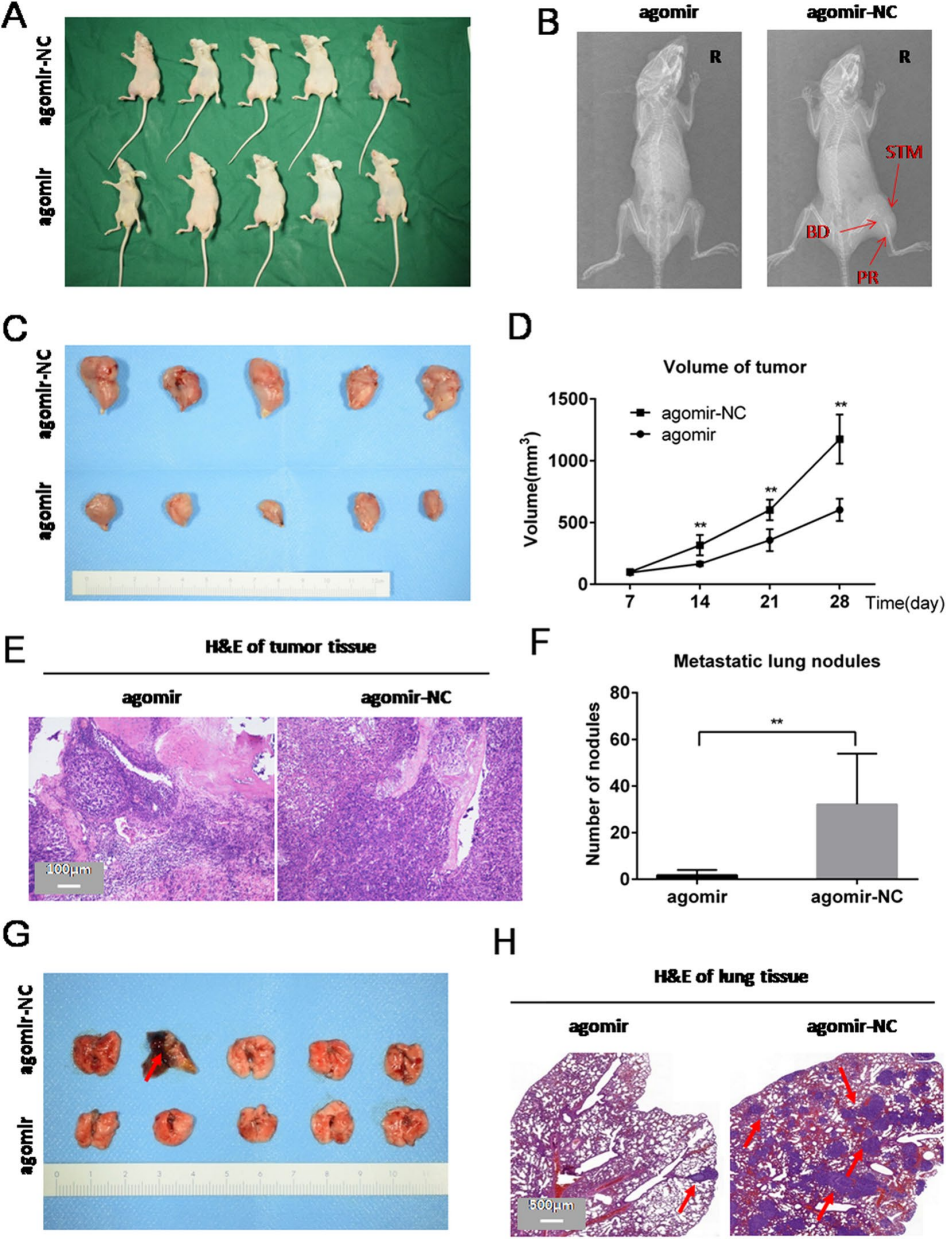
MiR-302b inhibited osteosarcoma tumour growth and lung metastasis in nude mice. (**A**) Orthotopic tumour transplantation was conducted by injecting 143B cells into the tibial periosteum of nude mice (n = 5). At three weeks after treatment with miR-302b agomir (agomir) or agomir negative control (agomir-NC), mice were sacrificed. (**B**) Orthotopic tumours were identified by X-ray imaging in which bone destruction (BD), periosteal reaction (PR) and soft tissue mass (STM) could be found. (**C**) Representative tumours are shown. (**D**) Representative photos of H&E-stained sections of orthotopic tumours formed in the two groups. (**E**) Growth curve of tumour volumes. (**F**) Quantification of lung metastatic nodules in each group. (**G**) Representative photos of lung tissue isolated from sacrificed mice in each group. **(H**) Representative photos of H&E-stained sections of lung tissue from each group and arrows represent the metastatic nodules. (*P < 0.05, **P < 0.01).

To explore the effect of miR-302b on tumourigenicity *in vivo*, we conducted orthotopic tumour transplantation by injecting 143B cells into the tibia periosteum of nude mice. Seven days later, a miR-302b agomir was injected intratumourally, and the animals were monitored closely for tumour growth for 3 weeks. The success of the orthotopic tumour was determined by X-ray images in which bone destruction, periosteal reaction and soft tissue mass could be assessed (Fig. 7A,B,C). As shown in Fig. 7C,D, treatment with miR-302b agomir resulted in a reduction of more than 40% in tumour volume compared with the control group. The mice were anaesthetized, and their tumours and lungs were dissected and stained with H&E to evaluate tissue morphology. H&E staining results of tumour tissue in the miR-302b agomir-treated group showed a larger area of necrosis than was found in the control group (Fig. 7E). These results suggested that miR-302b- agomir inhibited osteosarcoma tumour growth *in vivo*. Compared with the control group, the number of lung metastasis nodules was significantly reduced in the miR-302b agomir treatment group, as evidenced by H&E staining and macroscopic observation of lung specimens (Fig. 7F,G,H).

## Discussion

Dysregulation of miRNAs has been thoroughly documented in almost all types of human malignancy^13,14^. Improved investigation on the gene interaction mediated by miRNAs may provide further potential biomarkers and therapeutic targets for patients suffering from cancer. In the current study, preliminary evidence was presented about the negative regulatory role of miR-302b in osteosarcoma invasion and migration via targeting Runx2.

MiR-302b is differentially expressed in several human cancers. Specifically, miR-302b is down-regulated in breast cancer^15^, hepatocellular carcinoma^16^, pleural mesothelioma^17^, and gastric cancer^18^ and acts as a tumour-suppressing, anti-oncogenic miRNA. Our results revealed that miR-302b expression level was lower in osteosarcoma cell lines than in osteoblastic cell lines. Consistent with our previously published microarray data^10^, miR-302b was significantly down-regulated in osteosarcoma tissues compared with the normal adjacent bone tissues in the present study. Low expression of miR-302b was associated with distant and lymph node metastasis in osteosarcoma. Furthermore, from a gain-of-function study, miR-302b dramatically induced apoptosis and cell cycle arrest and suppressed cell proliferation, invasion, and migration *in vitro*. Likewise, treatment with miR-302b agomir significantly reduced the proliferation and lung metastasis of osteosarcoma *in vivo*. Therefore, our results presented a more comprehensive understanding of the potential tumour suppressor role of miR-302b in osteosarcoma progression.

Local invasion and metastasis are the main causes of death in osteosarcoma patients^8^. Therefore, it is essential to determine the mechanisms governing the metastasis of osteosarcoma to develop novel therapeutic strategies for osteosarcoma patients. In general, miRNAs fulfil their biological function via regulating their target genes. Several target genes of miR-302b have been identified, including E2F1^15^, E2F3^19^, Mcl-1^20^, CDK2^18^, AKT2^16^ and ErbB4^21^. Based on bioinformatics analysis, Runx2 was further identified as a putative target gene of miR-302b and that was verified by luciferase reporter assays in present study. Additionally, in miR-302b-overexpressing and miR-302b-downregulated osteosarcoma cells, the mRNA and protein levels of Runx2 were inhibited and promoted, respectively.

Runx2, belonging to the Runt domain family of transcription factors, is required for the differentiation of mesenchymal cellsalong the osteoblast lineage^22,23^. Sadikovic *et al*.^24^ reported that RUNX2 was significantly overexpressed in human osteosarcoma tumours and osteosarcoma cell lines compared with their counterparts. In addition, Runx2 is critical for osteosarcoma metastasis. Reduced Runx2 protein expression has been directly linked to upregulated miRNAs in human osteosarcoma cells compared with mesenchymal progenitor cells^25^. These genetic studies suggest that Runx2 may play a critical role in osteosarcoma oncogenesis on account of its significant function in regulating osteoblast differentiation and cell invasion and migration. Indeed, several miRNAs have been identified to directly target Runx2 mRNA to generate their biological effects. For example, Qin *et al*. showed that miR-455 restrains the cell migration and invasion activity of human hepatocellular carcinoma by targeting Runx2^26^. Consistently, miR-217 represses cell proliferation and invasion by targeting Runx2 in human glioma^27^. Furthermore, in osteosarcoma, Runx2 is also targeted by several miRNAs including miR-34c^28^, miR-205^29^, miR-30a^30^, miR-23a^31^. Our data indicate for the first time that Runx2 is a functional target of miR-302b. Down-regulation of Runx2 resulted in significant repression of cell invasion and migration, mimicking the biological effect of miR-302b overexpression. Moreover, upregulation of Runx2 significantly rescued the repressive effect of miR-302b.

Taken together, our results show that decreased miR-302b expression in osteosarcoma is associated with tumour development and metastasis. Furthermore, miR-302b functions as a tumour repressor in the invasion and migration activity of osteosarcoma by downregulating Runx2. Accordingly, deeper clarification of the specific role of miR-302b is proposed in the interest of identifying a potential therapeutic target for osteosarcoma.

## Materials and Methods

### Cell lines and tissue specimens

The human osteosarcoma cell lines MG-63 and 143B, purchased from the China Centre for Type Culture Collection (Wuhan, China), were maintained in DMEM (Gibco, Shanghai,China) containing 10% FBS (Gibco, Scoresby, Australia) and 1% antibiotics (penicillin and streptomycin). Thirty-one pairs of osteosarcoma and adjacent noncancer bone tissue samples were collected from patients undergoing biopsy before the chemotherapy at the orthopaedics department of Zhongnan Hospital of Wuhan University. Tissue specimens were immediately flash frozen in liquid nitrogen and stored until RNA extraction. The study was approved by the Institute Research Ethics Committee of Zhongnan Hospital of Wuhan University. Informed consent was obtained from all participants and/or their legal guardians. The human sample collection and treatment for total RNA isolation in present study were carried out in accordance with the approved guidelines.

### Plasmids construction and transfection

MiR-302b minic (mimic), miR-302b inhibitor (inhibitor), miR-negative control, Runx2 expression vectors, siRNA-ctrl, and siRNA-Runx2 were purchased form Guangzhou Ribobio Co., LTD. Cells were transiently transfected with 50 nM mimics or 50 nM inhibitor using Lipofectamine 2000 (Invitrogen). After 24 hours of transfection, the transfection efficiency was verified by qRT-PCR and the subsequent experimental procedure was performed. Other transfections were also performed by using Lipofectamine 2000 according to the manufacturer’s instructions.

### Quantitative real-time PCR analysis

Total RNA was extracted from tumour specimens and sample cells using TRIzol (Invitrogen). cDNA was synthesized by reverse transcription from total RNA using a Prime Script RT Reagent Kit (Toyobo, Japan) following the manufacturer’s introductions. Quantitative real-time PCR was performed using SYBR® Green qPCR Master Mix (Thermo Fisher Scientific). The gene expression of mature miR-302b was normalized to U6 as an endogenous control, and the expression of Runx2 was normalized relative to the endogenous control of human glyceraldehyde-3-phosphate dehydrogenase (GAPDH). The relative expression of these genes was calculated using the 2^−△△Ct^ method^32^. The specific primers were as follows: miR-302b forward 5′-ATCCAGTGCGTGTCGTG-3′, reverse 5′-TGCTTAAGTGCTTCCATGTT-3′; U6 forward 5′-CTCGCTTCGGCAGCACATATACT-3′, reverse 5′- ACGCTTCACGAATTTGCGTGTC-3′; Runx2, forward 5′-TGACCAGTCTTACCCCTCCT-3′, reverse 5′-CTGAAGCACCTGAAATGCG-3′; MMP2 forward 5′- AGATCTTCTTCTTCAA GGACCGGTT-3′, reverse 5′-GGCTGGTCAGTGGCTTGGGGTA-3′; MMP-9, forward 5′- CGCTGGGCTTAGATCATTCC -3′, reverse 5′- AGGTTGGATACATCACTGCATTAGG-3′; MMP-13, forward 5′- CCTTCTGGTCTTCTGGCACAC-3′, reverse 5′- GGCTGGGTCACACTTCTCTGG-3′; MMP-14, forward 5′- AGCCCCGAAGCCTGGCTACA -3′, reverse 5′-GCCGCCCTCACCATCGAAGG -3′; VEGF, forward 5′- AGGAGGAGGGCAGAATCATCA-3′, reverse 5′- CTCGATTGGATGGCAGTAGCT -3′; GAPDH,forward 5′-TCCACCACCCTGTTGCTGTA-3′, reverse 5′-ACCACAGTCCATGCCATCAC-3′.

### CCK-8 assay

Cells were seeded in 96-well plates (Corning, USA) and cultivated for 24, 48, 72, or 96 hours after transfection. CCK-8 working solution was added to each well, followed by incubation at 37 °C for 2 hours. Absorbance was measured at 450 nm with a Microplate Autoreader (SpectraMax M2, Molecular Devices, Sunnyvale, CA, USA). Experiments were repeated three times.

### Apoptosis and cell cycle assay

For apoptosis detection, cells (1 × 10^5^cells per well) were seeded in 6-well plates. At 24 hours after transfection, the cells were stained with an AnnexinV/PI double staining kit (Biotyme) according to the manufacturer’s instructions. Apoptotic cells were detected by flow cytometry. For cell cycle analysis, transfected cells were detached by trypsin digestion and washed with ice-cold PBS. Then, the cells were resuspended and fixed in 80% ethanol for at least 8 hours at −20 °C. The cells were stained with 50 μg/ml propidium iodide (Keygen, Nanjing, China). Cell cycle distribution was analysed using a FACS Calibur (BD Bioscience, MA, USA).

### Transwell assay

Cell invasion was evaluated using a transwell assay performed according to the manufacturer’s instructions (BD, Biosciences). After 24 hours of transfection, cells were resuspended in serum- free medium. Then, cell suspension was added to the top chamber, which was coated in advance with 60 μl of Matrigel (dilution of 1:8), followed by incubation at 37 °C in a humidified incubator for 12 hours. The lower chamber was filled with DMEM medium containing 10% FBS. Cells that invaded through the transwell chamber were fixed with formaldehyde for 10 min, washed with PBS, stained with crystal violet (Sigma-Aldrich). The invasion ability of the cells was quantitated by counting cells in five different areas under a microscope.

### Wound healing assay

Cell migration ability was evaluated using a wound healing assay. Cells were seeded in 6-well plates and allowed to reach 80% confluence. A wound was artificially created by scratching the cell monolayer with a 200 μl pipette tip. Plates was washed with PBS to remove the detached cells and maintained in Opti-MEM I Reduced-Serum Medium (Gibco). Wound closure was observed at 0 and 24 hours, and the image was photographed using a microscope. The migration distance (MD) in each group was calculated according to the following equation: MD = the width of the scratch at 0 hours (Width_0h_) - the width of the scratch at 24 hours (Width_24h_). The MD of the control group was used as a reference. The relative cell migration ability was determined by the following equation: Relative cell migration ability = MD (experimental group)/MD (control group).

### Dual-luciferase reporter gene assay

A Dual-Luciferase Reporter Assay System (Promega) was used to detect the luciferase reporter gene. All procedures were performed as previously described^33^. The 3′-UTR reporter plasmids were synthesized by placing amiR-302b seed sequence containing a Runx2 3′-UTR fragment in the pmirGLO-REPORT Luciferase miRNA Target Expression Reporter Vector. The wild-type or mutant reporter vector was cotransfected into 143B cells in 96-well plates with 50 nmol/L miR-302b or 50 nmol/L miR-NC and pmirGLO plasmid via using Lipofectamine 2000. The reporter gene assays were conducted 24 hours after transfection using the Dual-Luciferase Reporter assay system following the manufacturer’s protocol. The normalized luciferase activity for each construct was compared with that of the pmirGLO Vector control. All experiments were performed at least three times.

### Western blot analysis

Sample cell and tissue specimens were lysed with RIPA solution containing protease inhibitor. The lysates, containing total protein, were centrifuged to remove the cellular debris. Protein concentrations were measured with the Bradford protein assay (Beyotime, China) following the protocol. Total protein samples were separated by 10% SDS-PAGE and transferred to a PVDF membrane (Millipore, Billerica, USA). The membrane was blocked using 5% skim milk in TBST solution for 2 hours at room temperature. After triplicate washes in TBST, the membrane was incubated with specific primary antibodies against Runx2, OPN, MMP-2, MMP-9, MMP-13, MMP-14 and VEGF (Abcam, Cambridge, UK) at 4 °C overnight. Then, the blot was washed with TBST three times for 15 min each and incubated with secondary antibodies (Abcam). GAPDH was used as an internal reference. Immunoreactive bands were developed with an enhanced chemiluminescence kit (Bio-Rad) following the manufacturer’s instruction and then detected with a ChemiDoc™ Imaging System (Bio-Rad).

### Orthotopic osteosarcoma xenograft tumour mouse model

The care and treatment procedures for the experimental animals in this study were in accordance with the ethical standards of the Declaration of Helsinki and the national and international guidelines of the People’s Republic of China. The study was approved by the ethical committee of Zhongnan Hospital of Wuhan University. Ten 4-week-old male nude mice (BALb/c) were obtained from Beijing HFK Bioscience Co., Ltd. (Beijing, China). Orthotopic osteosarcoma xenograft tumours were produced using a subperiosteal injection method based on the previously published literature^34,35^. Briefly, 143B cells (4–6 passages) were resuspended in PBS to a final concentration of 3 × 10^7^ cells/ml. Animals were anaesthetized by intraperitoneal injection of pentobarbital (80 mg/kg). 100 µl of prepared cell suspension containing 3 × 10^6^ cells was injected subperiosteally into the right proximal lateral tibia using a 25-gauge needle. The cell suspension was prevented from oozing out by applying gentle pressure to the injection point with cotton swab for 10 seconds. Then, the animals were divided into two groups: the miR-302b agomir treatment group and the agomir control group. Seven days after tumour inoculation, the xenograft tumours were approximately at 100 mm^3^. The miR-302b agomir or agomir control (5 nmol in 0.1 ml per mouse) was injected intratumourally every three days. Tumour growth was observed every day after tumour inoculation. A Vernier caliper was used to measure the length and width of the tumour. Tumour volume (mm^3^) was calculated using the following equation: length × width^2^ × 0.5. Digital plain-film X-ray images covering the entire affected hind limbs were taken by Digital Diagnost (Philips Healthcare, Netherlands) to evaluate the orthotopic tumour formed by the 143B cells. Typical images of osteolysis, osteosclerosis, periosteal reaction and soft tissue mass were regarded as the characteristics of osteosarcoma.

### Haematoxylin and eosin (H&E) staining

After being sacrificed, mice were anatomized to obtain the tumour tissue from the hind limbs and the lung tissue. All the tissue samples were fixed in paraformaldehyde, embedded in paraffin, and sectioned with a microtome into 5-μm-thick sections using routine methods. The sections were deparaffinized with xylene for 10 min and rehydrated with 100% ethanol, 96% ethanol, 80% ethanol, 70% ethanol, and H_2_O, 5 min for each step. The sections were stained with 10% haematoxylin (Sigma-Aldrich) for 7 min, washed with tap water for 10 min, and stained for 5 min in 1% eosin (Sigma-Aldrich) containing 0.2% glacial acetic acid. Then, the slides were dehydrated in ethanol with graded concentrations of 70%, 80%, 96%, and 100%, 2 min for each step, followed by 5–10 min in xylene. The sections were observed and photographed using a light microscope (Leica, Germany).

### Statistical analysis

All data were subjected to statistical analysis using SPSS 17.0. Measurement data are expressed as the mean ± SD from at least three independent experiments. Student’s *t*-test or one-way ANOVA was applied to determine the differences. *P* < 0.05 was considered statistically significant.
